# Significance of the broad non-bony attachments of the anterior cruciate ligament on the tibial side

**DOI:** 10.1038/s41598-022-10806-8

**Published:** 2022-04-27

**Authors:** Satoru Muro, Jiyoon Kim, Sachiyuki Tsukada, Keiichi Akita

**Affiliations:** Department of Clinical Anatomy, Tokyo Medical and Dental University (TMDU), 1-5-45 Yushima, Bunkyo-ku, Tokyo, 113-8510 Japan

**Keywords:** Anatomy, Musculoskeletal system, Ligaments

## Abstract

Knowledge of the anatomy of the anterior cruciate ligament (ACL) is important to understand the function and pathology of the knee joint. However, on the tibial side of ACL, its structural relationships with the articular cartilage and lateral meniscus remain unclear. Furthermore, conventional research methods are limited to analyzing the bone attachments. We provide a comprehensive, three-dimensional anatomical description of the tibial side of the ACL that questions the principle that “a ligament is necessarily a structure connecting a bone to another bone.” In our study, 11 knees from 6 cadavers were used for macroscopic anatomical examinations, serial-section histological analyses, and three-dimensional reconstructions. The attachments of the tibial side of ACL consisted of attachments to the bone (102.6 ± 27.5 mm^2^), articular cartilage (40.9 ± 13.6 mm^2^), and lateral meniscus (6.5 ± 4.6 mm^2^), suggesting that the ACL has close structural relationships with the articular cartilage and lateral meniscus. Our study demonstrates that the tibial side of the ACL is not attached to the bone surface only and provides new perspectives on ligamentous attachments. Considering its attachment to the articular cartilage would enable more accurate functional evaluations of the mechanical tensioning of the ACL.

## Introduction

Knowledge of the anterior cruciate ligament (ACL) anatomy is important for understanding its function and the possible pathologies that may involve this structure. ACL injuries can occur simultaneously with damage to the lateral meniscus (LM) and articular cartilage^1,2^. Furthermore, in a cohort study of risk factors and predictors of subsequent ACL injury after ACL reconstruction, so-called the MOON study, risk factors for poor outcomes at 10 years after reconstruction included LM procedures and articular cartilage lesions^3^. These functional and pathological relationships strongly suggest structural relationships between the ACL, LM, and articular cartilage.

The tibial side of the ACL is attached to the bony surface of the anterior intercondylar area^4,5^. The following details are already known: there are variations in the shape of the tibial attachment, such as elliptical, triangular, and C-shaped. The tibial attachment is anteriorly bordered by a bony ridge, and the area of the complete tibial attachment of ACL is approximately 110 mm^2^^6–13^. However, several studies have also reported a close relationship between the ACL and LM, indicating a structural “overlap”^7,11,12,14–17^. One study using high-resolution 3-T MRI identified an overlap between the ACL and anterior horn of LM in 61.9% of patients^7^. Although some anatomical studies have also suggested a blending between the fibers of ACL and LM^11,17^, other histological studies have contradicted this phenomenon^12,14^. In contrast, there are few reports on the structural relationship between the ACL and articular cartilage. Therefore, a detailed structural relationship between the ACL and LM, including the presence or absence of fiber blending, and the structural relationship between the ACL and articular cartilage remain unknown on the tibial side of ACL, which may be attached to structures other than the bone.

This study investigated whether the tibial side of ACL has non-bony attachments and aimed to clarify the presence, location, extent, and histologic characteristics of these attachments. We have previously investigated the structures of ACL and LM and their relationship through a combination of macroscopic anatomical examinations, histological analyses, and micro-CT analyses^15,18–21^. Micro-CT is an excellent method for analyzing bone morphology; however, there are limitations in its ability to analyze the cartilage and ligaments. Therefore, in this study, we have added new methods, including serial-section histology and three-dimensional reconstruction, to clarify the anatomical details of the tibial side of ACL and the structural relationships of ACL with LM and articular cartilage. We hypothesized that (1) the fibers of ACL would blend with that of LM, and (2) ACL would attach to the articular cartilage of the medial facet. Proving these hypotheses would contribute to understanding the pathogenesis of ACL injuries with simultaneous damage to the LM and articular cartilage and optimizing postoperative rehabilitation based on the functional linkage between the ACL and LM.

## Results

### Macroscopic anatomy

**Figure 1:**
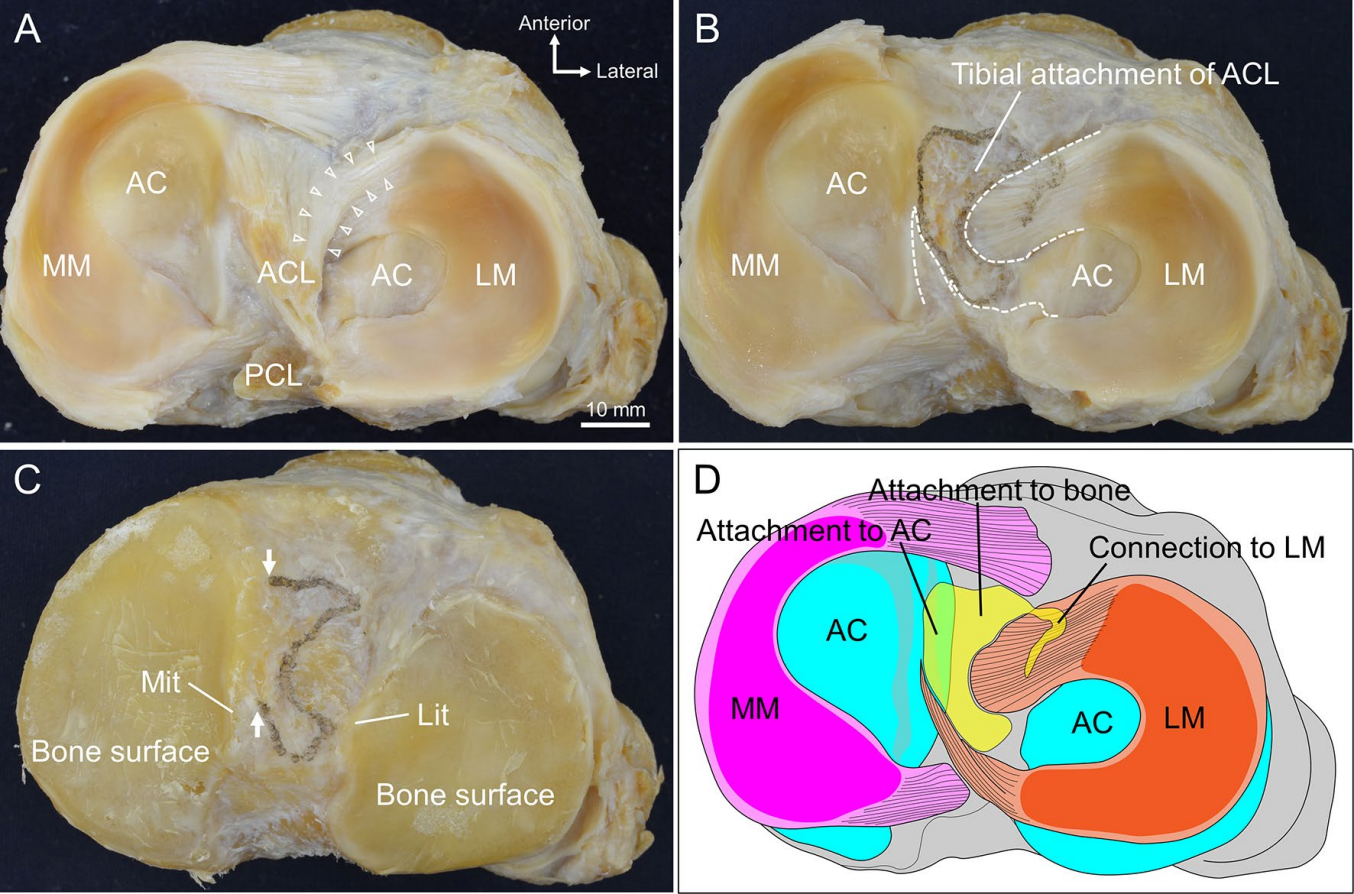
The attachment of the tibial side of ACL. (**A**) The superior aspect of the tibial side of the ACL. A few ACL fibers are connected to the anterior horn of LM (arrowheads). (**B**) After removing the ACL, while marking the margin of its attachment with a black pen. The lateral and posteromedial borders of the ACL attachment coincide with the edge of the anterior and posterior horns of LM, respectively. (**C**) After removing the menisci and articular cartilage. The marking line on the medial border of the ACL attachment disappeared (arrows), implying that the ACL is medially attached to the articular cartilage. (**D**) Summary of the attachments of the tibial side of ACL, comprising an attachment to the bone, articular cartilage, and LM. AC, articular cartilage; ACL, anterior cruciate ligament; Lit, lateral intercondylar tubercle; LM, lateral meniscus; Mit, medial intercondylar tubercle; MM, medial meniscus; PCL, posterior cruciate ligament.

The ACL was cut in half in the transverse section, and its tibial side was observed from its superior aspect (Fig. 1A). A few ACL fibers were connected to the anterior horn of LM (indicated by the arrowheads in Fig. 1A). The margin of the ACL attachment was marked with a black pen while each ACL fascicle was being removed individually (Fig. 1B). The concave lateral border of the ACL attachment coincided with the edge of the anterior horn of LM, whereas the posteromedial border of the ACL attachment coincided with the edge of the posterior horn of LM. After removing the menisci, articular cartilage removal caused the disappearance of the marking line on the medial border of the ACL attachment (indicated by arrows in Fig. 1C), indicating that the ACL was medially attached to the articular cartilage. The attachments on the tibial side of ACL comprised areas of attachment to the bone and articular cartilage and an area that was continuous with the LM (connection to LM) (Fig. 1D). The total area of attachment on the tibial side of ACL was 151.9 ± 37.2 mm^2^, of which the area of attachment to the bone, articular cartilage, and LM was 102.6 ± 27.5 mm^2^ (67.5%), 40.9 ± 13.6 mm^2^ (26.9%), and 6.5 ± 4.6 mm^2^ (4.3%), respectively (Table 1).

**Table 1:** Areas of attachment of the tibial side of ACL (n = 10).

Areas of ACL attachment (tibial side)	Mean [SD], mm^2^	Percentage (%)	ICC	95% CI
Complete attachment	151.9 [37.2]	–	0.998	0.994–1
Attachment to bone	102.6 [27.5]	67.5	0.998	0.994–1
Attachment to AC	40.9 [13.6]	26.9	0.999	0.996–1
Attachment to LM	6.5 [4.6]	4.3	0.999	0.994–1
Attachment to TL	1.9 [5.2]	1.3	0.996	0.984–0.999

*AC* articular cartilage, *ACL* anterior cruciate ligament, *CI* confidence interval, *ICC* intraclass correlation coefficients, *LM* lateral meniscus, *TL* transverse ligament.

**Figure 2:**
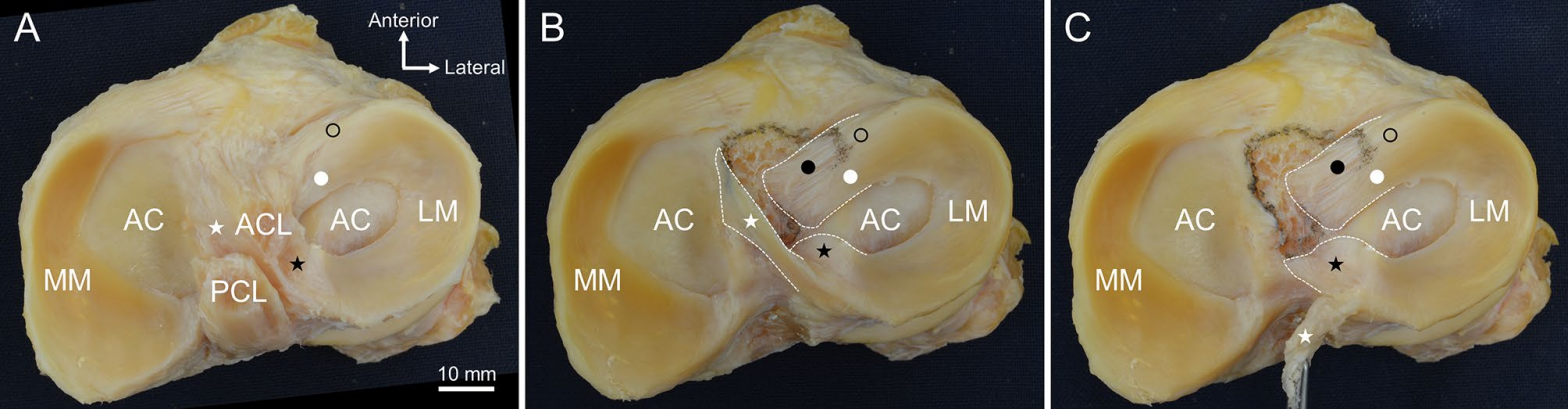
The relationship between the ACL attachment and the anterior and posterior horns of LM. (**A**) The superior aspect of the tibial side of ACL and LM. The superficial layer of the outer fibers of the anterior horn of LM (black circle) is connected to the ACL fibers. (**B**) After removing the ACL, while marking the margin of its attachment with a black pen. The deep layer of the outer (black dot) and inner fibers (white dot) of the anterior horn of LM extend into the intercondylar area and fit into the concave lateral border of the ACL attachment. The posteromedial crus (white star) is wrapped around the posteromedial side of ACL, and its attachment edge coincides with the medial border of the ACL attachment. (**C**) After removing the attachment of the posteromedial crus of the posterior horn of LM and flipping it posteriorly. The attachment edge of the anterolateral crus (black star) of the posterior horn of LM coincides with the posterior border of the ACL attachment. AC, articular cartilage; ACL, anterior cruciate ligament; LM, lateral meniscus; MM, medial meniscus; PCL, posterior cruciate ligament.

The arrangements of the anterior and posterior horns of LM were also observed with attention to their relationship with the tibial side of ACL (Fig. 2). Figure 2A shows the superior aspect before ACL removal, and Fig. 2B shows the superior aspect after ACL removal. The superficial layer of the outer fibers of the anterior horn of LM (indicated by the black circle in Fig. 2) was connected to the ACL fibers. The deep layer of the outer (indicated by the black dot in Fig. 2) and inner fibers (indicated by the white dot in Fig. 2) of the anterior horn of LM extended into the intercondylar area and fitted into the concave lateral border of the ACL attachment. In contrast, the posteromedial crus of the posterior horn of LM (indicated by the white star in Fig. 2) was attached to the medial intercondylar tubercle instead of the intercondylar area. In Fig. 2C, the attachment of the posteromedial crus of the posterior horn of LM (indicated by the white star in Fig. 2) was removed, and was flipped posteriorly. The attachment edge of the anterolateral crus (indicated by the black star in Fig. 2) of the posterior horn of LM coincided with the posterior border of ACL attachment, whereas the posteromedial crus (indicated by the white star in Fig. 2) was wrapped around the posteromedial side of ACL, with its attachment edge coinciding with the medial border of ACL attachment.

### Histology

**Figure 3:**
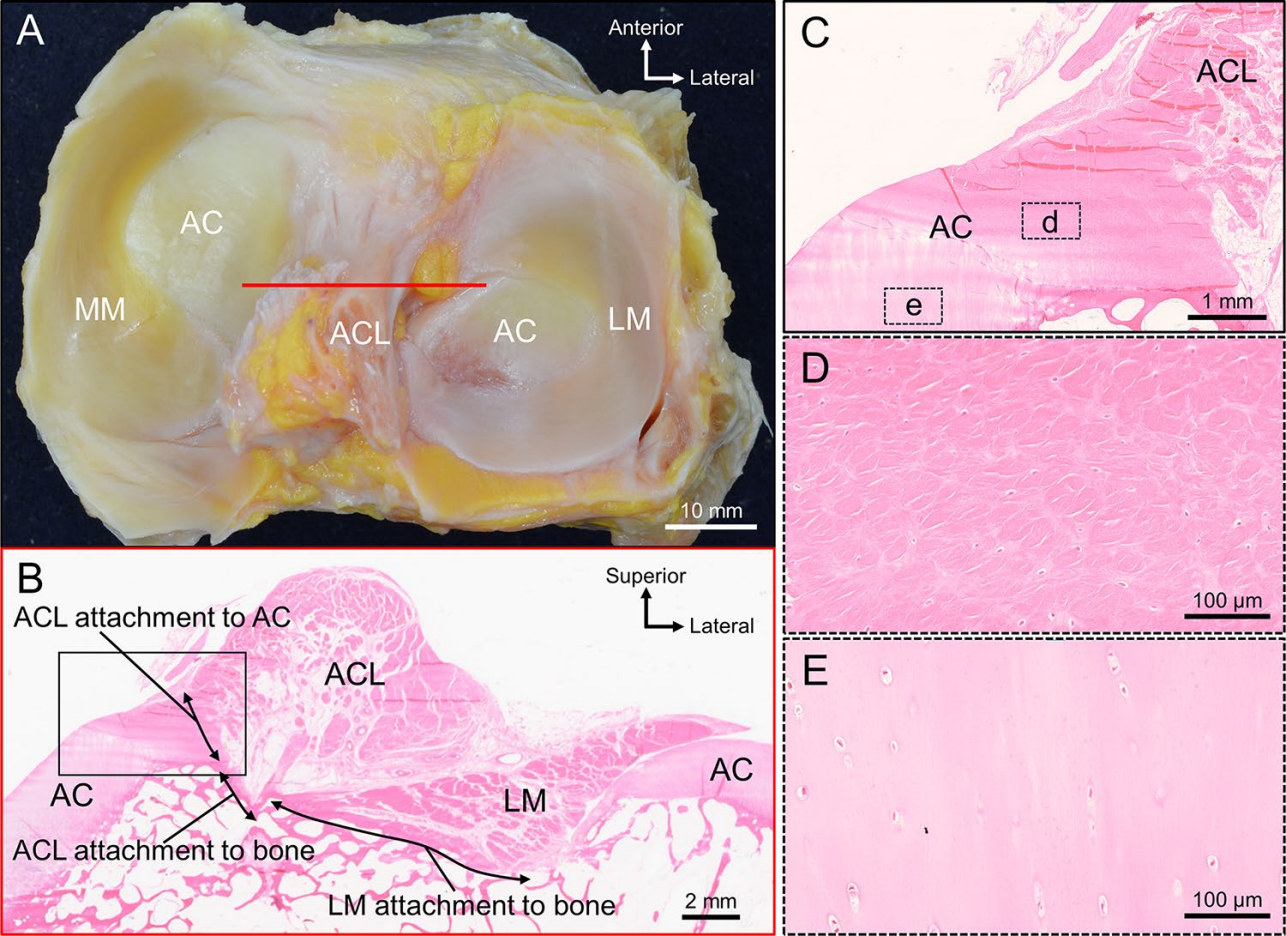
The attachment of ACL to the articular cartilage. (**A**) The superior aspect of the proximal side of the tibia, with the collection sites for the coronal sections indicated by a red line. (**B**) Coronal section of the red line in (**A**). The LM enters the intercondylar area from the lateral side and is broadly attached to the bone surface. ACL is narrowly attached to the bone surface, and the remaining part of the ACL is attached to the articular cartilage on the medial side. (**C**) A magnified view of the black rectangular space in (**B**). The attachment region of ACL to the articular cartilage is composed of a layer of fibrocartilage similar to the attachment region of ACL to the bone. (**D**) A magnified view of the fibrocartilage in (**C**). (**E**) A magnified view of the hyaline cartilage in (**C**). AC, articular cartilage; ACL, anterior cruciate ligament; LM, lateral meniscus; MM, medial meniscus.

The coronal section, taken from the red line, including the two intercondylar eminences in Fig. 3A, is shown in B. In the intercondylar area, LM entered from the lateral side and attached to the bone surface. LM was broadly attached to the bone surface of the intercondylar area, whereas ACL was narrowly attached (Fig. 3B). At the boundary between the LM and ACL attachments, a minute step was observed on the bone surface, and the inclination angle changed. The ACL attachment was localized to the medial side and consisted of attachments to the bone and articular cartilage. At the location of the ACL attachment, the surfaces of bone and cartilage were continuous, forming a steep, inclined surface facing laterally, with the ACL attached to this inclined surface (Fig. 3B). The attachment region of ACL to the articular cartilage was composed of a fibrocartilage layer (Fig. 3C–E), similar to the attachment region of ACL to the bone.

**Figure 4:**
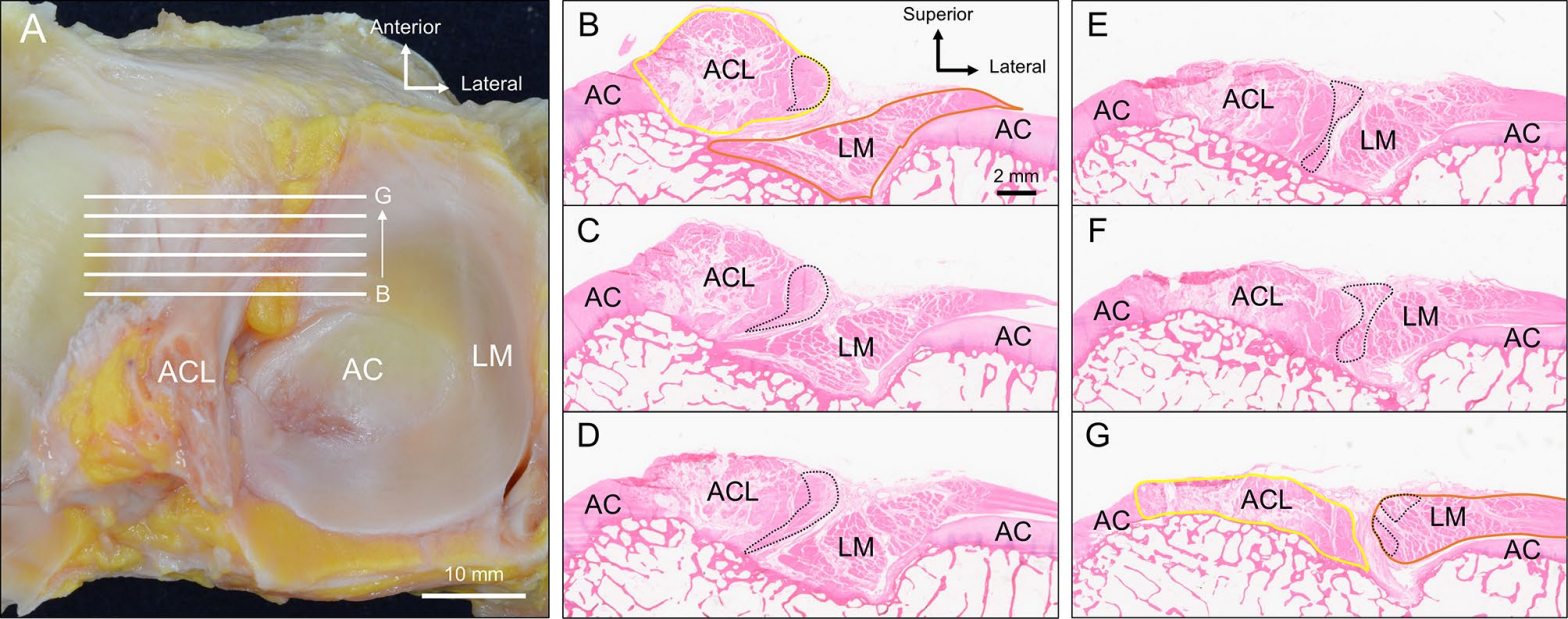
The blending of the ACL and LM fibers. (**A**) The superior aspect of the proximal side of the tibia with the collection sites for the coronal sections, indicated by white lines. (**B**–**G**) shows the serial, stepwise coronal sections of the white lines in (**A**) at 0.8 mm intervals. (**B**) The posterior-most section. The ACL and the anterior horn of LM are observed independently. (**C**–**F**) A few ACL fibers (dotted line) located on the lateral side are gradually displaced laterally while being separated from the ACL and approaching the LM. (**G**) The anterior-most section. These fibers (dotted line) are completely integrated with the LM. AC, articular cartilage; ACL, anterior cruciate ligament; LM, lateral meniscus.

The region from B to G (shown by the white lines in Fig. 4A) was observed on serial coronal sections at 0.2 mm intervals. Some of these sections are shown in Fig. 4B–G as serial stepwise coronal sections at 0.8-mm intervals, with Fig. 4B being the most posterior section and Fig. 4G being the most anterior section. As shown in Fig. 4B, the ACL and anterior horn of LM were observed independently. A few ACL fibers located on the lateral side (indicated by the dotted line in Fig. 4B–G) were gradually displaced laterally from Fig. 4C–F. Through this progression, divergent fibers from the ACL were observed to approach LM. Finally, as shown in Fig. 4G, these fibers were completely integrated with the LM. This histological examination showed that some ACL fibers were blended into the fibers of the anterior horn of LM.

### Three-dimensional reconstruction

**Figure 5:**
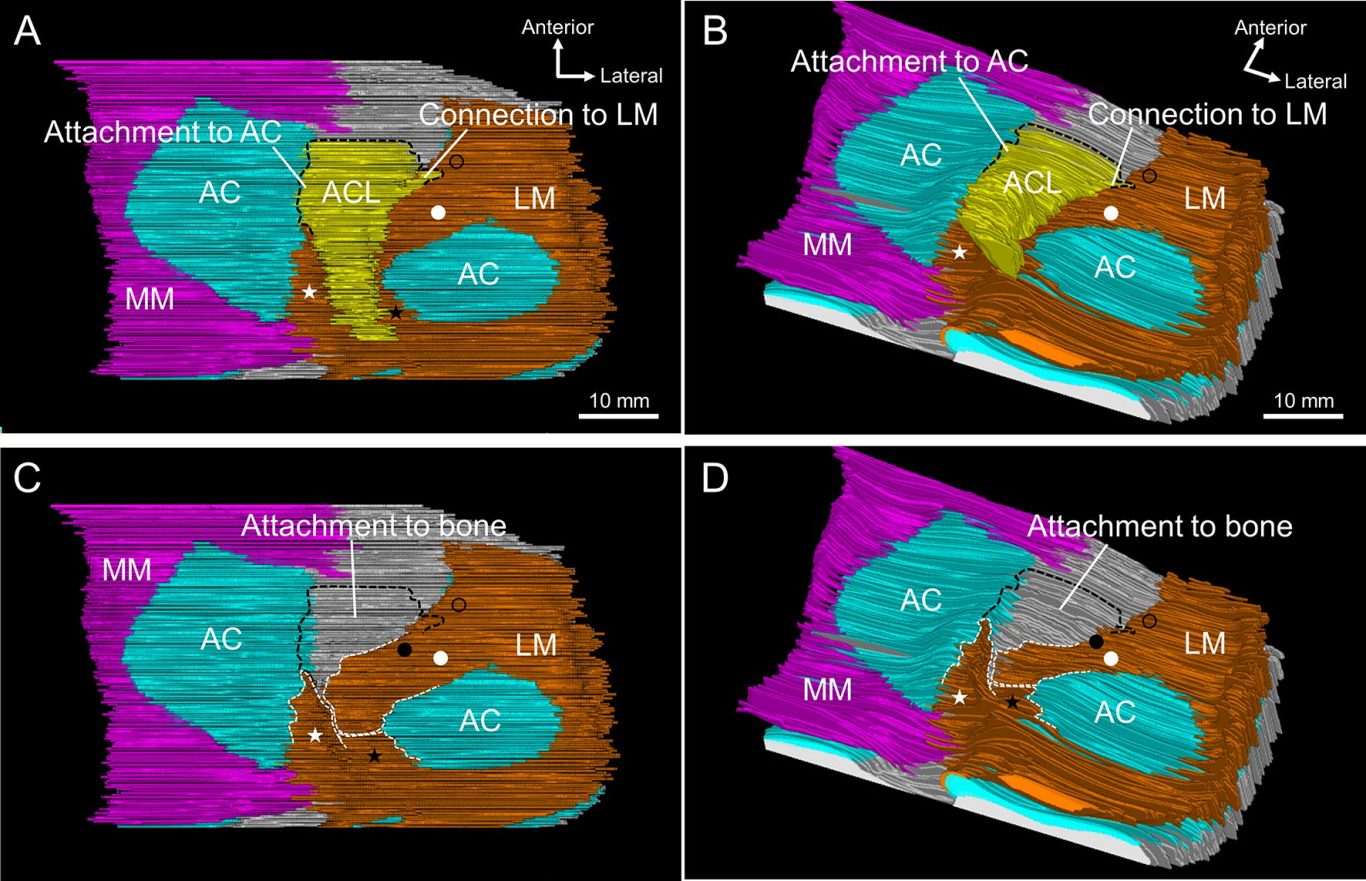
Three-dimensional relationships of the ACL with the AC and LM. (**A**) The superior aspect of the tibial side of the ACL and surrounding structures, reconstructed based on the serial histological sections (interval = 0.2 mm). The ACL is medially attached to the articular cartilage and laterally connected to the superficial layer of the outer fibers of the anterior horn of LM (black circle). (**B**) The posterolateral aspect of (**A**). (**C**) After removal of the ACL from (**A**). The deep layer of the outer (black dot) and inner fibers (white dot) extend into the intercondylar area. (**D**) The posterolateral aspect of (**C**). The articular cartilage in the area where the ACL is attached has an inclined surface facing laterally. AC, articular cartilage; ACL, anterior cruciate ligament; LM, lateral meniscus; MM, medial meniscus.

Figure 5 shows a three-dimensional image reconstructed using the serial histological sections. The medial side of ACL was attached to the articular cartilage, and a few ACL fibers located on the lateral side were connected to the anterior horn of LM (Fig. 5A,B). After the ACL was removed from the images, it was observed that the articular cartilage in the area where the ACL was attached had an inclined surface facing laterally (Fig. 5C,D). Additionally, ACL was connected to the superficial layer of the outer fibers of the anterior horn of LM (indicated by the black circle in Fig. 5). The deep layer of the outer (indicated by the black dot in Fig. 5) and inner fibers (indicated by the white dot in Fig. 5) extended into the intercondylar area (Fig. 5C,D).

## Discussion

This study clarified that the attachments on the tibial side of ACL are not only with the bone surface but also with the LM and articular cartilage. The ACL was also observed to be broadly attached to the articular cartilage on the medial side, and a few ACL fibers located on the lateral side appeared blended with those of the anterior horn of LM. Thus, ACL attachments on the tibial side comprise attachments to the bone, articular cartilage, and LM. These findings demonstrate that the tibial side of ACL has close structural relationships with both the articular cartilage on the medial side and LM on the lateral side, which would contribute to elucidating the traumatological mechanism of ACL injuries with simultaneous damage to the LM and articular cartilage. Furthermore, the detailed anatomy of the tibial side of ACL revealed by this study would provide an anatomical basis for orthopedic surgeons to design more rational ACL reconstruction procedures.

The tibial side of ACL is generally described as attaching only to the bone surface^4,5^. However, some structures, including the cruciate ligaments, menisci, and articular cartilage, are adjacent to each other in a narrow area of the proximal surface of the tibia within the knee joint. Therefore, these structures may have a direct structural relationship. To the best of our knowledge, the structural relationship between the ACL and articular cartilage has not yet been reported. Conventionally, it was believed that the tibial side of ACL did not attach to the articular cartilage, and only its attachment to the bone surface was analyzed^7,10–14,16,20,22–41^. In contrast, several studies have reported the close relationship of the tibial side of ACL with LM, describing it as an “overlap,” “adjoin,” or “adjacent”^7,11,12,14–17^. These reports have suggested that the attachments on the tibial side of ACL are not limited to the bony surface, i.e., there may be attachments to non-bony structures. However, the presence or absence of blending between the ACL and LM fibers and of attachment between the ACL and the articular cartilage has not been clarified previously. In this study, histological observations and three-dimensional reconstructions revealed that a part of the ACL on the medial side was attached to the articular cartilage. Interestingly, ACL attachment to the articular cartilage occupied approximately 27% of the entire attachment area; therefore, articular cartilage appears to play an essential role as an attachment site for a part of the ACL. These findings may even question the previously held principle that “a ligament is a structure connecting a bone to another bone.” In addition, we histologically demonstrated the blending of ACL fibers and those of the anterior horn of LM through a detailed analysis of the serial sections. This blending of fibers guarantees structural continuity between the ACL and LM.

In terms of ACL attachment to the articular cartilage, it is interesting to focus on the positional relationship between the ACL and anterior horn in the intercondylar area. In the intercondylar area, the region where the exposed bony surface is limited, and the articular cartilage is located on both sides. Because the anterior horn of LM extends into this intercondylar region, the ACL and LM appear to compete for the limited surface area of the bone. Therefore, the stronger the insertion of the LM, the narrower the area of ACL attachment to the bone surface. The ACL is attached to the articular cartilage on the medial side, probably to secure the attachment range. Our present study demonstrated that the ACL attachment to the articular cartilage was mediated by the fibrocartilage similar to the one that attaches ACL to the bone, indicating that the ACL is firmly attached to the articular cartilage and not just bordering on it. These findings suggest that the mechanical force of the ACL is applied to the articular cartilage. Therefore, it would not be sufficient to discuss the attachment of the tibial side of ACL solely to the bone. When considering the mechanical tensioning of the ACL, it is necessary to consider its attachment to the articular cartilage. Although we showed that approximately 27% of the attachment area on the tibial side of ACL was to the articular cartilage, it should be noted that this ratio does not denote the number of fibers or the ratio of the mechanical tension rather, the surface area. In the future, we plan to analyze the number and density of fibers and the tension applied to the attachment area.

The blending of ACL and LM fibers revealed in this study may be derived from the embryological closeness between the ACL and LM. During the development of the knee joint, both ACL and LM begin to form in O'Rahilly stages 21 and 22 and become well-advanced during week 9^42,43^. It is speculated that these two structures, developing simultaneously in the joint, develop in continuity. In addition, this continuity, accomplished through the blending of fibers, is believed to be functionally important for two reasons: (1) the formation of a hoop structure and (2) mutual support and control of the ACL and LM. First, LM forms approximately four-fifths of a circle, i.e., an incomplete circle^4^. Through the blending of fibers, the ACL and LM appear to form a perfect circular “hoop structure” to obtain higher stability. The idea that ACL and LM operate as a complex has been proposed in previous studies^11,16^, and this study provides an anatomical and histological basis for this concept. Second, the ACL and LM probably influence each other through the blending of fibers between both structures. In the posterior part of the knee joint, the meniscofemoral ligament connects the posterior cruciate ligament to the posterior horn of LM and is believed to support the posterior cruciate ligament by minimizing the displacement caused by posteriorly directed forces on the tibia and controlling the motion of the posterior horn of LM during knee flexion^44^. In contrast, in the anterior part of the knee joint, our study showed that the blending of ACL and LM fibers exists as a structure connecting ACL and LM, and its function is similar to the function of the meniscofemoral ligament in the posterior part of the knee joint.

Our study had some limitations. First, the sample size was relatively small. Second, the ages of the materials were skewed because the cadavers used in this study were those of elderly adults with an average age of > 60 years. Finally, this study was purely anatomical; therefore, we could not provide quantitative measurements related to ACL force. Techniques, such as contrast-enhanced X-ray CT and phase contrast-enhanced synchrotron X-ray micro-tomography, may be useful for the quantitative evaluation of the ACL fascicle alignment^45–47^. In the future, a biomechanical study may provide additional information regarding the mechanisms of ACL injury.

## Conclusion

This study clarified that the attachments on the tibial side of ACL consist of attachments to the bone, articular cartilage, and LM. The area of attachment to the cartilage is as wide as 27% of the total area, indicating that it is an important attachment for ACL. The ACL attachment to LM involves a blending of the fibers, enabling the ACL-LM complex to form a highly stable hoop structure. Our findings provide a new perspective on ligamentous attachments.

## Methods

### Preparation of cadaveric specimens

A total of 11 knees from 6 Japanese cadavers (3 males and 3 females; mean age at death, 65.3 years; range 52–74 years) were donated to our department. The donation document format was congruent with the Japanese law entitled “The Act on Body Donation for Medical and Dental Education” (Act No. 56 of 1983). Before their deaths, all donors voluntarily declared that their remains would be donated as materials for education and study. At that time, the purpose and methods of using body donor corpses were explained, and informed consent was obtained. After their death, we also explained this informed consent to the bereaved families and confirmed that there was no opposition. All cadavers were fixed by arterial perfusion of 8% formalin and preserved in 30% alcohol. Cadavers with a history of knee abnormalities were not included in the study. Study approval was obtained from the Board of Ethics at the Tokyo Medical and Dental University (approval number: M2018-243). All methods were carried out in accordance with the relevant guidelines and regulations.

### Macroscopic anatomy

Ten knees were used for macroscopic examinations. Specimens with a marked degeneration of the ACL fibers, trauma, or disease involving the proximal tibia were excluded. After removing the skin and subcutaneous soft tissues, the patellar tendon was cut from the tibial tuberosity. We used the proximal tibial surface where the ACL and menisci remained. A middle transverse section of the ACL was performed, and all other supporting tissues, including the posterior cruciate ligament, capsule, and collateral ligament, were separated at the attachment to the tibia. The superior aspect of the ACL and LM were then observed with special attention to the attachment between the structures. While removing each ACL fascicle individually, the margin of the ACL attachment was marked with a black pen. After all ACL fibers were removed, the positional relationship between the ACL attachment and the anterior and posterior horns of LM were observed. Thereafter, menisci and articular cartilage were removed, and the remaining marked area was observed. We measured the area of ACL attachment using ImageJ (version 1.52; National Institutes of Health, Bethesda, Maryland, United States of America)^48^. The photographs of the specimens taken with a ruler were imported into ImageJ software, and the magnification was standardized with the “Set Scale” command. Considering that the ACL attachment area is not a simple oval, the area selection tool of "Polygon" and the analyzing tool of "Measure (Area)" were used for the measurement.

### Histology

The remaining knee was used for the histological examination to prepare the serial coronal sections. The proximal tibial surface with the ACL and surrounding structures were harvested as a tissue block (anteroposterior diameter, 40 mm; height, 20 mm; width, 65 mm) using a diamond band pathology saw (EXAKT 312; EXAKT Advanced Technologies). The tissue block was fixed by immersing it in 10% formalin for 24 h. The block was then decalcified in Plank-Rychlo solution (AlCl_3_:6H_2_O 126.7 g/L, HCl 85 mL/L, HCOOH 50 mL/L) for 5 days and dehydrated (70% ethanol, 80% ethanol, 90% ethanol, 100% ethanol twice, xylene twice) for at least 24 h in the solution at each step^15^. These immersion and fixation processes took approximately six times longer than usual, given the size of the tissue block. Thereafter, the block was embedded in paraffin over 5 days while changing the paraffin solution three times. The paraffin-embedded tissue block was serially sectioned in the coronal plane into 5-μm-thick specimens at 0.2-mm intervals. Histological sections were then stained with hematoxylin and eosin using the following protocol: (1) deparaffinization and rehydration (xylene for 5 min, 4 times; 100% ethanol for 3 min, 4 times; 90% ethanol for 3 min; 70% ethanol for 3 min; tap water for 3 min; and deionized H_2_O for 1 min), (2) hematoxylin staining (hematoxylin applied for 10 min; rinse with tap water; immersed 3 times in acid ethanol to de-stain; tap water rinse for 20 min to allow the stain to develop; and rinse with deionized water for 1 min), and (3) eosin staining and dehydration (eosin applied for 5 min; immersed in 100% ethanol for 3 min, 4 times; and xylene for 5 min, 4 times). The stained specimens were scanned as whole slides using a high-quality scanner (GT-X980, EPSON, Japan), and then local high magnification digital images were acquired using a digital slide scanner (NanoZoomer-SQ C13140, HAMAMATSU, Japan).

### Three-dimensional reconstruction

The attachments on the tibial side of ACL were analyzed using computer-assisted, three-dimensional reconstructions made from serial histological coronal sections obtained from the histological examination described above. All 160 serial sections were scanned, and the structures (ACL, posterior cruciate ligament, LM, medial meniscus, articular cartilage, and bone [tibia]) were traced and colored. Section sequences were reconstructed using SrfII software (ver. R.11.00.00.0-H, Ratoc, Tokyo, Japan, http://www.ratoc.com/eng/index.html) using a technique that was similar to what was used in our previous reports (Three-dimensional reconstruction of fibrous tissue of the knee joint and musculature of the pelvic floor)^49–51^.

### Statistical analysis

Measurements of the macroscopic examinations were performed by two measurers (S. M. and J. K.), and an intraclass correlation coefficient (ICC) was calculated to test the reliability of the measurements. The measurement values were the areas of the following attachments on the tibial side of ACL: complete attachment and attachment to the bone, articular cartilage, LM, and transverse ligament; these values measured by S. M. and by J. K. were compared. A score above 0.75 was considered to indicate excellent agreement. Statistical analyses were performed using R software for Windows (version 4.1.0; R, The R Foundation for Statistical Computing, Vienna, Austria)^52^, with the level of significance set at a *p*-value of < 0.05.

### Statement

All methods in the study were carried out in accordance with the relevant guidelines and regulations.

### Ethical approval

The study was approved by the Ethics Committee of Tokyo Medical and Dental University (Approval Number: M2018-243).

## Data Availability

The data that support the findings of this study are available from the corresponding author upon reasonable request.
